# *Pleurotus eryngii* Genomes Reveal Evolution and Adaptation to the Gobi Desert Environment

**DOI:** 10.3389/fmicb.2019.02024

**Published:** 2019-09-03

**Authors:** Yueting Dai, Lei Sun, Xiaolei Yin, Meng Gao, Yitong Zhao, Peisong Jia, Xiaohui Yuan, Yongping Fu, Yu Li

**Affiliations:** ^1^Engineering Research Center of Chinese Ministry of Education for Edible and Medicinal Fungi, Jilin Agricultural University, Changchun, China; ^2^International Cooperation Research Center of China for New Germplasm and Breeding of Edible Mushrooms, Jilin Agricultural University, Changchun, China; ^3^Institute of Plant Protection, Xinjiang Academy of Agricultural Sciences, Xinjiang, China; ^4^School of Computer Science and Technology, Wuhan University of Technology, Wuhan, China

**Keywords:** *Pleurotus eryngii* var. *eryngii*, *Pleurotus eryngii* var. *ferulae*, King Oyster, genome sequencing, differentiation, genetic variation

## Abstract

*Pleurotus eryngii* (King Oyster) is one of the most highly prized edible mushrooms. Among the diverse varieties within *P. eryngii*, *P. eryngii* var. *eryngii* is the commonest one, with a worldwide distribution, while *P. eryngii* var. *ferulae* is only distributed in Europe and China, and is especially adapted to the Gobi Desert in Xinjiang Autonomous Region of China. However, little is known about the genome-wide pattern of evolution and adaptation to the divergent environments of *P. eryngii*. Here, we present the high-quality genome sequences of *P. eryngii* var. *eryngii* strain PEE81 originating from Europe and *P. eryngii* var. *ferulae* strain PEF12 originating from the Gobi Desert of China. The assembled genome sizes of PEE81 and PEF12 were 53.6 and 48.0 Mbp, respectively, which are larger than other reported genomes in the genus *Pleurotus*. We propose that the selective amplification of long terminal repeat (LTR) retrotransposons increases the genome size of the genus *Pleurotus*, and may play a key role in driving their rapid species diversification. Molecular clock analyses of five *Pleurotus* species, namely PEE81, PEF12, *P. tuoliensis, P. ostreatus* and *P.* cf. *floridanus* suggest that the divergence estimates of the genus *Pleurotus* over time scales ranged from ∼4 to ∼38 million years ago (Mya), and PEE81 and PEF12 diverged at ∼13 Mya. The whole genome resequencing of 33 geographically diverse strains of *P. eryngii* var. *eryngii* and var. *ferulae* was then performed and the genome variation among and within these two populations were investigated. Comparative analyses of these two populations detected several candidate genes related to stress responses and DNA repair that are putatively involved in adaptation to the Gobi Desert environment. These findings offer insights into genome evolution of the genus *Pleurotus* and provide valuable genomic resources for King Oyster mushroom breeding.

## Introduction

*Pleurotus eryngii* (Basidiomycota, Agaricales) fungi naturally grow on the roots of Apiaceae plants such as *Eryngium* spp. and *Ferula* spp., and are generally distributed in Europe, Asia and Africa (Zervakis and Balis, 1996; Zervakis et al., 2001, 2014). *P. eryngii sensu stricto* composed of at least five varieties such as var. *eryngii*, *ferulae*, *elaeoselini*, *thapsiae*, and *tingitanus*, which significantly differ in their habitat distribution, host, and morphology (Zervakis et al., 2001, 2014; Zhao et al., 2016). Among them, *P. eryngii* var. e*ryngii* and var. *ferulae* have been commercialized worldwide and given the commercial name as King Oyster (Moonmoon et al., 2010). *P. eryngii* var. e*ryngii* typically grows on the roots of *Eryngium* spp., *Peucedanum* spp., and *Opopanax chironium* and is distributed in a broad range across Europe, Central Asia, and Africa. Whereas *P. eryngii* var. *ferulae* only grows with *Ferula* and is distributed in Europe and China, especially being restricted to the Gobi Desert in Xinjiang, China. Moreover, *P. eryngii* var. *eryngii* has a brown cap of fruiting body, while var. *ferulae* is white to brown (Kawai et al., 2008; Zervakis et al., 2014; Yang R.H. et al., 2016; Zhao et al., 2016). The phylogenetic relationships of *P. eryngii* varieties have been revisited using ITS, EF-1α, RPB2, RPB1, and IGS sequences (Zervakis et al., 2014; He et al., 2016; Zhao et al., 2016). The results indicate that *P. eryngii* var. e*ryngii* in Europe and var. *ferulae* in Europe and China are sister groups and belong to two different evolutionary lineages, which might be due to geographic isolation. Therefore, additional studies are needed to better understand the species formation, evolutionary patterns, and genetic differentiation of the different *P. eryngii* varieties.

With the rapid development of high-throughput sequencing technologies, it is necessary to perform a systematic analysis of *P. eryngii* at the whole-genome level. Until now, four *Pleurotus* species have had their whole genome sequenced, including *P. eryngii* var. e*ryngii* (Yang R.H. et al., 2016; Zhang Z. et al., 2018), *P. ostreatus* (Qu et al., 2016; Wang et al., 2018), *P.* cf. *floridanus* (Riley et al., 2014; Alfaro et al., 2016; Castanera et al., 2016; Li et al., 2019), and *P. tuoliensis* (Gao et al., 2018; Zhang Z. et al., 2018). The genome sizes of *Pleurotus* species ranges between 34.3 and 49.9 Mbp and the number of predicted protein coding genes is 10,936–14,443. In addition, the whole genome sequences from two cultivated strains of *P. eryngii* var. e*ryngii* have been reported, but their genome sizes are slightly different (43.7 and 49.9 Mbp) (Yang R.H. et al., 2016; Zhang Z. et al., 2018). Furthermore, Zhang Z. et al. (2018) revealed the large differences in the types and quantity of carbohydrate-active enzymes in *P. eryngii* var. e*ryngii* and *P. ostreatus.* Specifically, *P. eryngii* var. e*ryngii* contains more genes encoding enzymes involved in the degradation of lignocellulose, such as AA1, AA7, AA9, and GH7 (Zhang Z. et al., 2018). However, the whole genome of *P. eryngii* var. *ferulae* has not been sequenced.

In this study, we performed *de novo* genome sequencing of two wild stains of *P. eryngii* using the single-molecule real-time (SMRT) sequencing platform, including *P. eryngii* var. *eryngii* PEE81 originating from Europe and *P. eryngii* var. *ferulae* PEF12 originating from the Gobi Desert of China. Following that, the HiSeq X-ten sequencing platform was used for the whole genome resequencing of 33 *P. eryngii* strains. The specific objectives were the following: (1) explore the genome evolution, genomic features and genome variation of *P. eryngii* var. *eryngii* originating from Europe and *P. eryngii* var. *ferulae* from China; and (2) detect the population genetic diversity, genetic differentiation, and environment adaptations of these two *P. eryngii* varieties.

## Materials and Methods

### Strains Used for Genome Sequencing

The protoplast-derived monokaryons of PEE81 from Europe and PEF12 from China were isolated according to Dai et al. (2017), which were used for the subsequent whole genome sequencing. Dikaryotic mycelia of PEE81 and PEF12 were cultured in a liquid Malt Yeast Extract Glucose (MYG) medium for 7 and 10 days (d), respectively. These mycelia were filtrated and washed with 0.6 M D-mannitol (DINGGUO, Beijing, China), and then incubated with 2.0% lywallzyme (Guangdong Institute of Microbiology, Guangzhou, China) for 150 min at 27°C. The filtrated protoplasts were cultured on the regeneration media (MYG solid medium with 0.6 M D-mannitol per liter) at 24°C. The regenerated monokaryons were determined after checking the number of nuclei using a microscope (Carl Zeiss AG, Jena, Germany) with fluorescent staining. Thirty-seven strains were used for the whole genome resequencing using the HiSeq X-ten platform, including 19 *P. eryngii* var. *eryngii* collected from Europe, China and Japan, and 14 *P. eryngii* var. *ferulae* and four *P. tuoliensis* collected from Xinjiang Autonomous Region in China (Supplementary Table S1). Each strain was cultured on MYG solid culture medium containing cellophane for 10 d. A Genomic DNA Kit (CWBIO, Beijing, China) was used to extract the genomic DNA for each strain. Agarose gel electrophoresis (0.6%), Qubit 3.0, and NanoDrop 2000 were used to examine the genomic DNA quality and concentration. All strains were maintained in the Engineering Research Center of Chinese Ministry of Education for Edible and Medicinal Fungi, Jilin Agricultural University (Changchun, China).

### *De novo* Genome Sequencing, Assembly, and Annotation

The 20 kb libraries were constructed for PEE81 and PEF12, respectively. The *de novo* whole genome sequencings of these two strains were then carried out using the Sequel platform of Pacific Biosciences (PacBio) (Sossah et al., 2019; Wang et al., 2019). SMARTdenovo^1^ was used for the *de novo* assembly of PEE81 and PEF12 genomes. Core Eukaryotic Genes Mapping Approach (CEGMA) (Parra et al., 2007) and Benchmarking Universal Single-Copy Orthologs (BUSCO) (Simão et al., 2015; Waterhouse et al., 2018) were used to assess the integrity of the assembled PEE81 and PEF12 genomes. The accession numbers of the genome data uploaded to GenBank are SZVJ00000000 (PEE81) and SPUN00000000 (PEF12).

Repeat elements were identified using a combination of *de novo* and homology-based approaches with RepeatModeler and RepeatMasker, respectively. Tandem repeats were identified using Tandem Repeats Finder (TRF)^2^. The microsatellite loci in the two genomes were identified by MISA tool^3^ (Fu et al., 2017). Gene predictions were conducted by combining *de novo* prediction and homology information. Four kinds of software including Augustus (Stanke et al., 2006), SNAP (Korf, 2004), GeneScan (Burge and Karlin, 1997) and GlimmHmm (Majoros et al., 2004) were used for *de novo* predictions. For homologous protein mapping, proteomes from four fungi were aligned to PEE81 and PEF12 genomes using tBLASTn and GeneWise (Birney et al., 2004), including *P.* cf. *floridanus* (Qu et al., 2016), *Agaricus bisporus* (Morin et al., 2012), *Schizophyllum commune* (Ohm et al., 2010) and *Coprinopsis cinerea* (Stajich et al., 2010). Finally, all gene models produced by *de novo* prediction and protein homology search were integrated using GLEAN (Elsik et al., 2007). For non-coding RNA, miRNAs and snRNAs were detected using Rfam database (Gardner et al., 2009). tRNA loci were detected using tRNAscan-SE (Lowe and Eddy, 1997). Ribosomal RNA (rRNA) was detected combined homologous BLASTn searches and RNAmmer (Lagesen et al., 2007). These putative protein-coding genes were functional annotated using National Center for Biotechnology Information (NCBI) non-redundant database (Nr), Eukaryotic Clusters of Orthologous Groups (KOG), SwissProt, Translated EMBL Nucleotide Sequence Data Library (TrEMBL), InterProScan, Gene Ontology (GO), Kyoto Encyclopedia of Genes and Genomes (KEGG)^4^ databases. Annotation of carbohydrate-active enzymes (CAZyme) was carried out with dbCAN2 meta server (Zhang H. et al., 2018). In addition, to more accurately perform the comparative genomic analysis in the genus of *Pleurotus*, we re-annotated the reported whole-genome sequences of *Pleurotus* on NCBI using the same parameters as for PEE81 and PEF12, including *P. tuoliensis* (Yang R.H. et al., 2016; Zhang Z. et al., 2018), *P. ostreatus* (Qu et al., 2016; Wang et al., 2018), and *P.* cf. *floridanus* (Riley et al., 2014; Alfaro et al., 2016; Castanera et al., 2016; Li et al., 2019).

### Evolution and Comparative Genomic Analysis

The protein-coding sequences from 10 fungal species were used for gene family analysis by OrthoMCL software (Li et al., 2003), including the above-mentioned five *Pleurotus* and five fungal species reported on NCBI with fossil records namely *Coniophora puteana* RWD-64-598 SS2 (Floudas et al., 2012), *Serpula lacrymans* S7.9 (Eastwood et al., 2011), *C. cinerea* okayama7#130 (Stajich et al., 2010), *Laccaria bicolor* S238N-H28 (Martin et al., 2008), and *S. commune* H4-8 (Ohm et al., 2010). The extracted single-copy genes were used to construct phylogenetic trees using RA × ML software (Stamatakis, 2014) based on the maximum likelihood (ML) method. The divergence times were estimated using MCMCtree in the PAML software (Sanderson, 2003). The calibration points of fossil time were according to Floudas et al. (2012). The number of expanded and contracted gene families in the 10 fungai species were predicted by Computational Analysis of gene Family Evolution (CAFE) software (De Bie et al., 2006). MCScan software^5^ was used to test the syntenic regions (cscore = 0.99) in PEE81 and PEF12.

### Population Genomic Analysis of *P. eryngii*

The high-quality clean reads from the 37 strains used in whole genome resequencing were aligned to the reference genome PEF12 using Burrows-Wheeler Aligner (BWA) (Li and Durbin, 2009) and SOAPaligner (Li et al., 2008). SAMtools software (Li et al., 2009) was used to statistics the mapping rate, sequencing depth and coverage rate. GATK software (Depristo et al., 2011) was used for detecting single nucleotide polymorphisms (SNPs) and insertion-deletions (InDels) in each strain. ANNOVAR software (Wang et al., 2010) was used for the annotation and statistical analysis of the distribution of variable regions in each strain. The high-quality population SNP set was then used to construct the phylogenetic tree of the 33 *P. eryngii* strains with four *P. tuoliensis* strains as the outgroup, using MEGA software (Kumar et al., 2016).

### Genome-Wide Selection Test of the Two *P. eryngii* Populations

The 19 *P. eryngii* var. *eryngii* and 14 var. *ferulae* strains were set as two populations. PoPoolation2 software (Kofler et al., 2011) and Sliding Window Algorithm were used to calculate the fixation index (F_ST_) for the two populations with a sliding window size of 5,000 bp and step size of 500 bp, and then Z transformation of F_ST_ (Z*Fst*) were carried out. A Z*Fst* > 4 was used as the threshold value to identify selected regions. Functional enrichment analysis was conducted on the genes in the selected regions using GO and KEGG database.

## Results

### Genome Sequencing and Assembly of *P. eryngii* var. *eryngii* and var. *ferulae*

We *de novo* sequenced the genomes of the monokaryon strains of *P. eryngii* var. *eryngii* PEE81 (hereafter, PEE81) originating from Europe and *P. eryngii* var. *ferulae* PEF12 (hereafter, PEF12) originating from the Gobi Desert of China using a PacBio Sequel system. The 4.13 Gb (∼84.40×) and 4.05 Gb (∼77.02×) single-molecule long reads were generated for PEE81 and PEF12, respectively. The *de novo* assembled genome sizes were estimated to be 53.6 Mb with contig N50 of 3.2 Mb for PEE81 and 48.00 Mb with contig N50 of 2.3 Mb for PEF12. Two genomes both possessed ∼49% GC content (Table 1). The vast majority of core eukaryotic genes (96%) and single-copy orthologs (90–96%) were aligned to these two *P. eryngii* genomes using CEGMA and BUSCO, respectively. These results indicated that we obtained the high-quality reference genomes for *P. eryngii*.

**TABLE 1:** Genome assembly and annotation results of the five *Pleurotus* species.

**Accession**	***P. eryngii* var. *eryngii***	***P. eryngii* var. *ferulae***	***P. tuoliensis***	***P. ostreatus***	***P.* cf. *floridanus***
Genome size (Mb)	53.6	48	43.4	34.7	34.3
Number of contigs	48	51	35	199	12
N50 (Mb)	3.2	2.3	2.7	1.1	3.3
GC content (%)	49.3	49.9	50.2	50.5	50.9
Repeat abundance (%)	32.8	33.3	26.2	11.6	11.5
LTR abundance (%)	31.9	32.3	25.4	11.1	11.0
Number of genes	12372	11515	10891	10723	10567
Average gene length (bp)	1849	1881	1889	1945	1967
Average number of exons per gene	5.5	5.6	5.7	5.9	6
Average exon length (bp)	243	253	243	249	248
Average intron length (bp)	116	102	106	95	94

### Gene Predictions and Annotations of *P. eryngii* var. *eryngii* and var. *ferulae*

To explore the structural and functional characteristics of the PEE81 and PEF12 genomes, we predicted and annotated the repeats, coding genes, and non-coding genes in the two genomes using a combination of *de novo* prediction and homologous-sequence alignment. The contents of the repeat sequences were similar in the PEE81 (18 Mb, 33%) and PEF12 (16 Mb, 33%) genomes. Among them, Long terminal repeats (LTRs) were the major type in both PEE81 (17 Mb) and PEF12 (16 Mb). In addition, the number of simple sequence repeats (SSRs) were 1,684 in PEE81 and 1,464 in PEF12, respectively. Among these SSR, the trinucleotide repeats (TNRs) were the most abundant type in both PEE81 (101) and PEF12 (951), followed by dinucleotide repeats (DNRs) (50, 390), hexanucleotide repeats (HNRs) (66, 65), tetranucleotide repeats (TTNRs) (61, 37), and pentanucleotide repeats (PNRs) (38, 21). We then successfully designed 1,684 and 1,464 SSR primer pairs for PEE81 and PEF12, respectively, using Primer 3. These newly developed primers can be used for further QTL mapping for the agronomic traits of *P. eryngii*.

We predicted 12,372 and 11,515 protein-coding genes with an average length of 1,843 bp and 1,881 bp in the PEE81 and PEF12 genomes, respectively. The average exon and intron lengths were 243 bp and 116 bp for PEE81, and 253 bp and 102 bp for PEF12 (Table 1). Among them, 11,436 (92%) and 10,719 genes (93%) genes were annotated for PEE81 and PEF12, respectively, by the eight databases, including NCBI Nr and Nt, KOG, SwissProt, TrEMBL, InterPro, GO and KEGG databases. In addition, the annotation results for non-coding RNAs showed that the PEE81 genome contained 266 transfer RNAs (tRNAs), 93 ribosomal RNAs (rRNAs), 15 small nuclear RNAs (snRNAs), and 27 micro RNA (miRNAs). Likewise, the PEF12 genome contained 183 tRNAs, 43 rRNAs, 17 snRNAs, and 49 miRNAs.

After alignment with the dbCAN server, we found that the total number and subtypes of the annotated genes encoding CAZyme gene families were similar between PEE81 (547) and PEF12 (539). Among them, there were 218 genes encoding glycoside hydrolases (GHs), 28 genes encoding polysaccharide lyases (PLs), 77 genes encoding glycosyltransferases (GTs), 69 genes encoding carbohydrate esterases (CEs), 98 genes encoding auxiliary activities (AAs), and 61 genes encoding carbohydrate binding modules (CBMs) in the PEE81 genome. Likewise, there were 214 GHs, 23 PLs, 68 GTs, 63 CEs, 94 AAs, and 73 CBMs in the PEF12.

### Evolution Analysis

**FIGURE 1:**
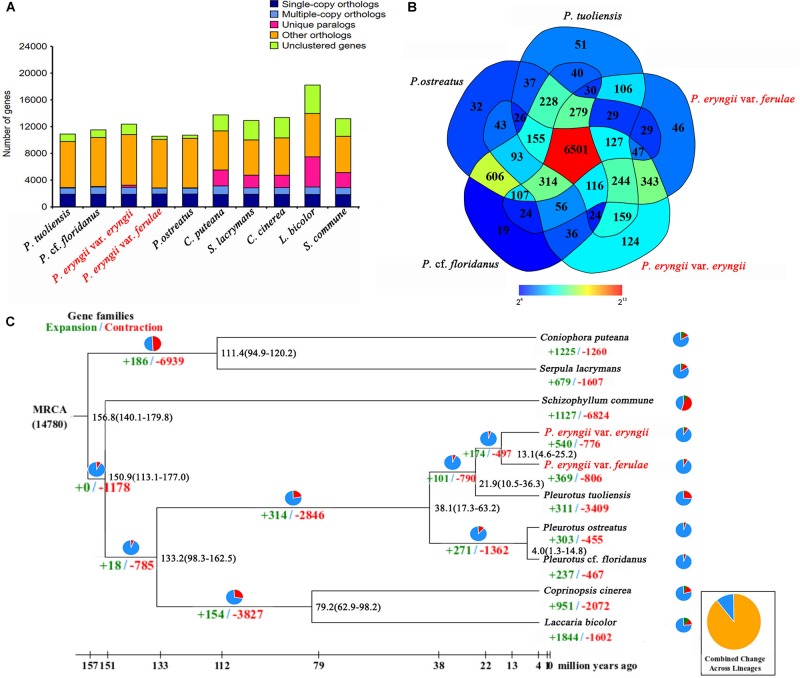
Comparative genomic analysis of *P. eryngii.* **(A)** Comparison of orthologous genes among the genomes of 10 fungal species. **(B)** Unique and shared gene families in the genomes of *P. eryngi*i var. e*ryngii*, var. *ferulae*, *P. tuoliensis*, *P. ostreatus* and *P.* cf. *floridanus*. The number of unique and shared gene families is shown in each of the diagram components. **(C)** Numbers on the nodes represent the divergence times. The number of expanded (green) and contracted (red) gene families in each lineage is shown on the corresponding branch. MRCA, most recent common ancestor.

To further perform comparative genomic analysis between *P. eryngii* and the other three reported species in the genus *Pleurotus* (*P. tuoliensis*, *P. ostreatus, P.* cf. *floridanus*), we used the same method combing with the uniform reference gene set used for the annotations of PEE81 and PEF12 to re-annotate three reported genomes to avoid systematic biases using the different methods. The detailed annotation results are shown in Table 1. The assembled PEE81 and PEF12 genomes were larger than the reported *Pleurotus* genomes. The repeat sequence contents of the two *P. eryngii* genomes and the closely related species (33% in PEE81 and PEF12, and 26% in *P. tuoliensis*) were higher than that of *P. ostreatus* and *P.* cf. *floridanus* (12%), especially LTR retrotransposons. In addition, a total of 6,501 homologous gene families and 5,228 single copy genes (Figure 1A) were shared by these five *Pleurotus*, and 124, 46, 51, 32 and 19 gene families were specific to the PEE81, PEF12, *P. tuoliensis*, *P. ostreatus*, and *P.* cf. *floridanus*, respectively (Figure 1B).

To further estimate the genome evolution of the genus *Pleurotus*, a total of 1,369 single-copy orthologous genes shared in 10 fungal species were used to construct a phylogenetic tree. The results showed that *P. eryngii* var. *eryngii* and var. *ferulae* were clustered with *P. tuoliensis* as a single branch that then clustered with *P. ostreatus* and *P.* cf. *floridanus*, which were separated into a clade based on the genus. The estimated divergence time between the genus *Pleurotus* and *C. cinereus* and *L. bicolor* is ∼133.2 million years ago (Mya). The species in the genus *Pleurotus* diverged between 4 to 38 Mya. The estimated divergence time of the ancestors of *P. tuoliensis* and the two *P. eryngii* is ∼22 Mya, and the diverged time between PEE81 and PEF12 is ∼13 Mya (Figure 1C).

Native selective pressures could lead to gene expansion and contraction during species evolution. A total of 540 and 776 gene families underwent expansion and contraction, respectively, in PEE81, whereas the number of expanded gene families in PEF12 was relatively lower (369) and the number of contracted gene families was higher (806). GO and KEGG analysis showed that these expanded gene families in the two genomes were both mainly associated with carbohydrate metabolic process, hydrolase activity, transmembrane transport, kinase activity, and transferase activity. It is worth noting that there were more expanded genes in PEF12 than PEF81 involving in carbohydrate and nitrogen metabolism, such as trehalose, xylulose catabolic process, fructose 6-phosphate, nitrogen compound, organonitrogen compound and cellular macromolecule metabolism, as well as cellular response to heat. In addition, there were some transport proteins associated with fungal parasitism and saprophytism, such as major facilitator superfamily, ATP-binding cassette, and mitochondrial carrier family, that also underwent expansion in these two genomes. The contracted genes in the PEE81 genome were mainly associated with DNA repair protein and leucyl-tRNA synthetase, while those in PEF12 mainly participate in the response to oxidative stress, oxidation-reduction process, and metabolic process.

### Comparative Genomic Analysis of *P. eryngii* var. *eryngii* and var. *ferulae*

**FIGURE 2:**
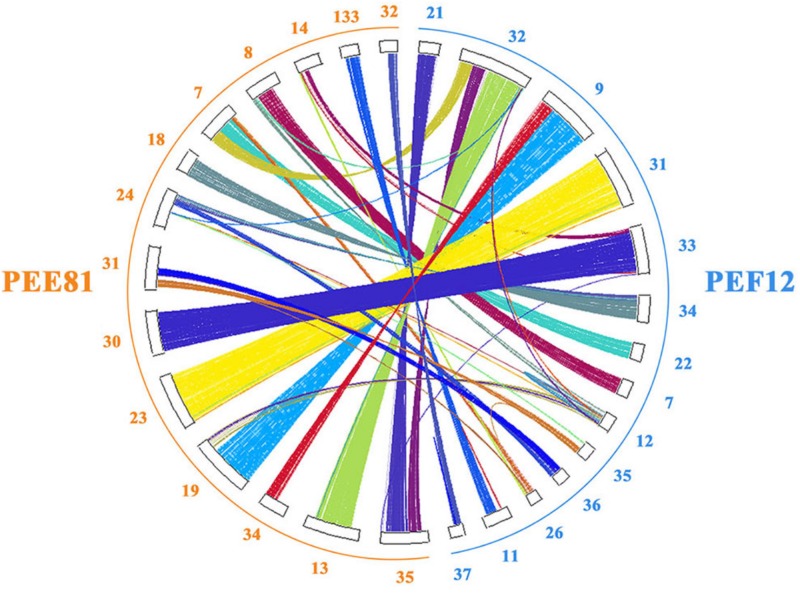
The genome colinearity analysis of the PEE81 and PEF12 using the protein-coding genes.

We identified 8,018 gene families shared in PEE81 and PEF12, and 221 and 96 unique gene families in PEE81 and PEF12, respectively. Functional enrichment analysis was then carried out on the unique gene families. The results showed that the specific genes in the PEE81 genome were mostly associated with MAP kinase phosphatase, transcription factor STE12, glutathione S-transferase, ornithine carbamoyltransferase, serine/threonine-protein kinase SRPK3, transcriptional regulator, GH47, and carbohydrate metabolic process, while genes encoding SRPK3, PL3, and hydrolase activity were specific for PEF12. Furthermore, we performed the colinearity analysis of these two genomes and found 1,295 highly syntenic regions (42 Mb) between them (Figure 2). We also detected some inversions and rearrangements occurred in the homologous regions. For example, a large inversion in contig13 of PEE81 and contig32 in PEF12, and a rearrangement in contig13 and contig35 of PEE81 and contig32 of PEF12 were seen. In addition, the two genomes possess their own unique regions, such as contig24 and contig31 in PEE81 and contig11 and contig37 in PEF12. The genes located in non-syntenic regions may be associated with their morphological formation and environmental adaptation.

### Population Genomic Analysis of *P. eryngii*

**FIGURE 3:**
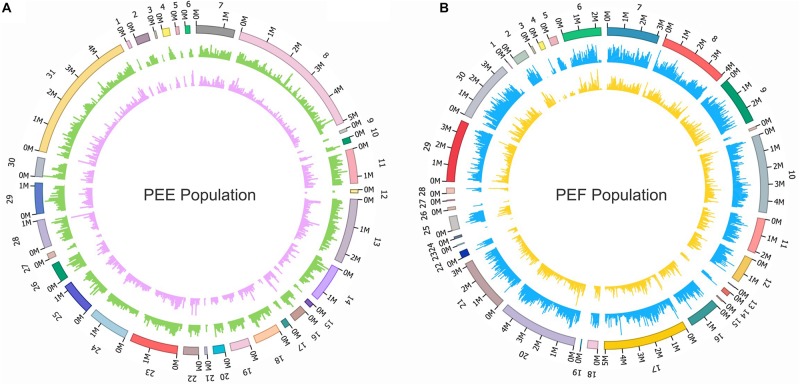
Circos plot of the SNPs and InDels distributions in *P. eryngii* var. e*ryngii* and var. *ferulae* populations. **(A)** The SNPs and InDels distributions of 19 *P. eryngi*i var. e*ryngii* strains using the reference genome PEE81. **(B)** The SNPs and InDels distributions of 14 *P. eryngi*i var. *ferulae* strains using the reference genome PEF12. Outside to inside of concentric circles show assembly scaffold number, SNPS distribution and density, InDels distribution and density (window size = 50 kbp).

We performed the whole genome resequencing of 19 *P. eryngii* var. *eryngii*, 14 var. *ferulae*, and four *P. tuoliensis* strains (as outgroup) to estimate the genome variations among and within these two populations and infer their population structure (Figures 3A,B). A total of 62 G clean data was yielded by the Illumina HiSeq platform. The average depth of each strain was up to 23×. We used the PEF12 genome as the reference genome to perform the genome alignments of the 37 strains. The coverage rates of these strains were up to 94–99% in *P. eryngii* var. *ferulae*, 87–89% in var. *eryngii*, and 74–75% in *P. tuoliensis* (Supplementary Table S2). The above data provides a preliminary proof that the phylogenetic relationship between *P. eryngii* var. *eryngii* and var. *ferulae* is closer than *P. tuoliensis*, which is consistent with previously identification results based on the morphology, ITS and other gene sequences, and SSR molecular markers.

A total of 1,219,829 high-quality SNPs were detected in the 37 strains after quality filtering using the PEF12 genome as a reference genome. Among them, 436,384 (35.8%) SNPs were located within exons, followed by intergenic regions (289,719, 23.8%), upstream of the gene (202,820, 16.6%), downstream of the gene (187,234, 15.3%), and introns (170,111, 13.9%). Out of the SNPs located in exons, there were 180,438 (41.7%) nonsynonymous SNPs and 252,531 (58.3%) synonymous SNPs, which resulted in a nonsynonymous/synonymous ratio of 0.7. Moreover, we identified 119 large (>100 bp) and 130,775 small (<50 bp) InDel among the 37 strains. Among them, 36,672 (27.8%) InDels were located in upstream of the gene, followed by intergenic regions (34,475, 26.2%), downstream of the gene (32,942, 25%), introns (23,066, 17.5%), and exons (16,760, 12.7%). Overall, 82 and 18% of InDels were found in coding regions and non-coding regions, respectively.

**FIGURE 4:**
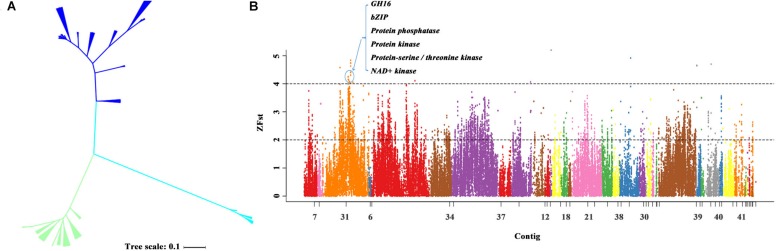
The genetic diversity and genome selection of *P. eryngii* var. e*ryngii* and var. *ferulae* populations. **(A)** The genetic tree of the 37 strains including 19 *P. eryngii* var. e*ryngii*, 14 var. *ferulae* strains, and four *P. tuoliensis.* **(B)** The genome selection region of *P. eryngii* var. e*ryngii* and var. *ferulae* populations using the reference genome PEF12.

We then constructed the phylogenetic tree for the closely related species of *P. eryngii* using the neighbor-joining method with *P. tuoliensis* as the outgroup. The 37 samples were divided into three distinct populations that is consistent with the corresponding species. The *P. eryngii* var. *ferulae* population from the Xinjiang Gobi desert of China is distinguishable from *P. eryngii* var. e*ryngii* population that possessed a relatively high level of genetic diversity (Figure 4A). Our results reveal the species growth with *Ferula* in the Gobi Desert in Xinjiang, China should belong to two different species (*P. tuoliensis* and *P. eryngii* var. *ferulae*) based on the genomic analyses.

To screen the genome regions of *P. eryngii* var. *ferulae* associated with adaptation to the Gobi Desert, we carried out the whole-genome genetic differentiation analysis of 19 *P. eryngii* var. e*ryngii* and 14 var. *ferulae* using the PEF12 genome as a reference genome. In the top 1% Z(F_ST_) region, 1.30 Mbp selected regions were obtained from the two populations. We further detected 18 genes within these regions mainly encoding protein phosphatase, protein kinase, NAD + kinase, bZIP transcription factor, transport protein,4′-phosphopantetheinyl transferase, and GH16 (Figure 4B).

## Discussion

### Genome Sequencing of *P. eryngii*

Living environment conditions such as Antarctic, desert and plateaus could drive species evolution (Kelley et al., 2013; Wu et al., 2014; Yang J. et al., 2016). Previous studies showed that there are large differences in the habitats, host and morphology of *P. eryngii* var. *eryngii* and var. *ferulae.* Therefore, *P. eryngii* is an ideal species for studying the environmental adaptive evolution and population differentiation of edible mushrooms. We hypothesized that *P. eryngii* needs a certain degree of genetic variation to adapt to the different environments. In this study, we used the Sequel platform for *de novo* whole-genome sequencing of the wild strains *P. eryngii* var. *eryngii* from Europe and var. *ferulae* from the Gobi Desert in Xinjiang, China. Compared with the two previously reported *P. eryngii* var. *eryngii* genomes of the cultivated strains, the number of contigs in the PEE81 genome was decreased to one-third and the N50 was increased to six times. In addition, we obtained the genome of *P. eryngii* var. *ferulae* for the first time. Our results not only provide a foundation for the development and utilization of germplasm resources of *P. eryngii*, but also provide the genomic resource for the further omics studies of the different *P. eryngii* varieties.

The expansion of repeated sequences, such as transposons, is one of the major factors for genome expansion. In this study, the repeated sequences in the genomes of *P. eryngii* and the closely related species *P. tuoliensis*, particularly LTRs, were significantly increased and their genomes were also larger compared with *P. ostreatus* and *P.* cf. *floridanus*. These results indicated large numbers of repeated sequences accumulated during the evolution of *P. eryngii*, that resulting in genome expansion. Within the *P. eryngii* and the closely related species *P. tuoliensis*, the genome size and proportion of repeated sequences showed a gradual increasing trend in *P. tuoliensis*, *P. eryngii* var. *ferulae*, and var. *eryngii.* The relative contraction of the *P. tuoliensis* and *P. eryngii var. ferulae* genomes may be due to the adaptive evolution in a harsh environment.

We then conducted the whole-genome collinearity analysis for PEE81 and PEF12, which demonstrated that some inversions or chromosomal rearrangements existed in homologous regions although they possessed highly syntenic regions. This suggests that the chromosomes might experience a series of fusions or breakages during the long evolutionary process of *P. eryngii*. Based on an assessment of the specific sites of homologous regions in watermelon, cucumber, and muskmelon chromosomes, Guo et al. (2013) suggested that complex changes in chromosomal structure constitute the main reason of this phenomenon.

### Molecular Basis for the Environmental Adaptation in *P. eryngii* var. *ferulae*

Previous research has shown that large number of organism explosion (such as flowering plants) during the Tertiary period (66–2.58 Mya), which experienced global warming and relatively high sea levels (Gradstein and Ogg, 2012). Based on the phylogenetic tree, we found that *Pleurotus* were differentiated into two clusters (*P. eryngii* and *P. ostreatus*) during the Tertiary period, indicating these two groups originated from the same ancestor and experienced parallel evolution and even species differentiation.

The expansion and contraction of a large number of gene families occurred during the evolution of *Pleurotus.* The expansion gene families in *P. eryngii* var. *ferulae* were significantly enriched in kinase activity, phosphatase activity, transmembrane transport, lignocellulose degradation, synthesize of secondary metabolites, carbon metabolism, nitrogen metabolism, and cellular response to heat. These genes might be related to the growth, development, and stress responses in organisms. For instance, serine/threonine protein kinase 1 (Ark1) plays an important role in spore development, stress response and pathogenicity of fungi by initiating reversible protein phosphorylation to activate protein cytoskeleton. Three expansions genes encoding Glutathione S-transferase occurred in *P. eryngii* var. *ferulae*, which played an important role in responding to abiotic stress, such as drought, chilling injury, and high salinity. All the above results might explain why *P. eryngii* var. *ferulae* can better adapt to the arid, cold, nutrient-deficient Gobi Desert in Xinjiang, China. In addition, the enrichment of genes associated with carbon metabolism, nitrogen metabolism, and cellular response to heat showed that *P. eryngii* var. *ferulae* can utilize more substrates to obtain nutrients for itself and have better tolerance to high temperatures, which might explain why it has a broad host range and does not require low temperature stimulation during fruiting body formation. Large numbers of transporter proteins were found among its expanded genes, of which the MFS and ABC transporter proteins were related to the infection of fungi on plants by resisting defensive compounds produced by plants and antibiotics that are toxic to fungi. While we identified some peptidase synthesis gene families, such as M28, M50, and M36, the number of genes was significantly lower than that in parasitic pathogen fungi (*Peltaster fructicola*) and no key genes such as carboxypeptidases were found, indicating a weak parasitic relationship between *P. eryngii* and host (Xu et al., 2016). Contracted gene families in the *P. eryngii* var. *ferulae* genome were significantly greater than the expanded gene families, which are mainly enriched in metabolism and cellular proliferation. This might be due to better adaption to the drought and poor nutrition environment in the Gobi desert in Xinjiang of China by maintaining a compact genome structure to reduce energy consumption.

### Population Genetic Analysis of *P. eryngii* Based on the Genome Sequencing

As genome research applied on edible mushrooms has existed for only several years from now, few population genomic studies based on the whole genome sequencing were available (*Lentinula edodes* and *Boletus edulis*) (Branco et al., 2015, 2017; Xiao et al., 2016). Our study constituted the first exploration of a population genomic analysis of *P. eryngii* and its closely related species. Based on the detected SNPs, we found that many of them were located in the CDS region, which is significantly higher than that in plants (such as tomato and cucumber) and animals (such as domestic duck and honey bee) (Lin et al., 2014; Chen et al., 2016). However, only minor parts of these SNPs lead to nonsynonymous mutations, which is significantly lower than the ratio in plants and animals. This might be due to varying degrees mutation of two nuclei in dikaryons, but these mutations did not cause variation in the genes’ function. Nonsynonymous mutation SNPs data provides a new resource for the biological research and breeding of the closely related species of *P. eryngii*, and there is also a need for further analysis in species evolution.

Based on the population selection analysis using PEF12 genome as the reference genome, we identified some genes that might be associated with the adaptation of *P. eryngii* var. *ferulae* to desert drought, intense sunshine and temperature variations. These genes mainly encode for protein phosphatase, kinase, transcription factor bZIP, and GH16. One example was OsbZIP71, a bZIP transcription factor, which was suggested to play an important role in abiotic stress in rice such as stress response to drought, salt and abscisic acid (ABA) stress (Liu et al., 2013). In addition, VaCPK20, as a serine/threonine-protein kinase, played a crucial role in drought and low temperature resistance in *Arabidopsis thaliana* (Dubrovina et al., 2015). Previous studies showed that GH16 was involved in drought and other stresses (Hrmova et al., 2007). In addition, we also identified some candidate genes using PEE81 genome as the reference genome (Supplementary Figure S1). We found some genes were the same with the result using PEF12 genome as reference, including genes encoding protein phosphatase, kinase and transcription factor. We also detected some genes associated with DNA repair and recombination and manganese peroxidase, indicating that these genes might also participate in the differentiation and adaptive evolution of *P. eryngii*. Therefore, we suggest that the different genomes of closely related species should be all chosen as reference genomes to conduct population genomic analysis, which could be more comprehensively characterize the genes associated with the genetic differentiation and adaption.

## Conclusion

In this study, we obtained the high-quality whole genome sequences of wild strains of *P. eryngii* from Europe and China. The comparative genomic and population genomic analysis results showed that: (1) a large number of repeated sequences accumulated during the evolution of *P. eryngii* and its closely related species compared with *P. ostreatus* and its closely related species, which resulted in the expansion of *P. eryngii* genome size; (2) the genus *Pleurotus* mainly evolved and diverged during the Tertiary period (66–2.58 Mya); (3) the evolutionary and population genetic analysis of gene families in *P. eryngii* var. *ferulae* showed that some genes might be associated with adaptation to aridity, large temperature changes, long sunshine durations, and other stresses in the Gobi Desert in Xinjiang, China. The genomic data produced in our study provide a valuable genetic resource for the development of new stress-resistant strains of *P. eryngii*.

## Data Availability

The raw datasets generated for this manuscript have been uploaded to GenBank, accession numbers SZVJ00000000 (PEE81) and SPUN00000000 (PEF12).

## Author Contributions

YF and YL conceptualized the study and provided funding. YD and YF wrote the manuscript and analyzed the data. LS performed the genome annotation. XY, YZ, MG, and PJ collected the sample and carried out the genome extraction. XY reviewed and edited the manuscript. All authors have read and approved the manuscript.

## Conflict of Interest Statement

The authors declare that the research was conducted in the absence of any commercial or financial relationships that could be construed as a potential conflict of interest.

## Footnotes

1 https://github.com/ruanjue/smartdenovo

2 http://tandem.bu.edu/trf/trf.html

3 http://pgrc.ipk-gatersleben.de/misa/misa.html

4 http://www.kegg.jp/

5 https://github.com/tanghaibao/jcvi/wiki/MCscan-(Python-version)

## Supplementary Materials

TABLE S1 Information of *Pleurotus eryngii and P. tuoliensis* strains used for whole genome resequencing.

TABLE S2 Summary of whole genome resequencing data of 33 *P. eryngii* and four *P. tuoliensis* strains using the reference genome PEF12.

FIGURE S1 The genome selection region of *P. eryngii* var. e*ryngii* and var. *ferulae* populations using the referencing genome PEE81.
