# Supplementary figures and images for: Pleurotus eryngii Genomes Reveal Evolution and Adaptation to the Gobi Desert Environment
Source: Front Microbiol. 2019 Sep 3;10:2024. doi: 10.3389/fmicb.2019.02024 (PMC6734163; doi:10.3389/fmicb.2019.02024)

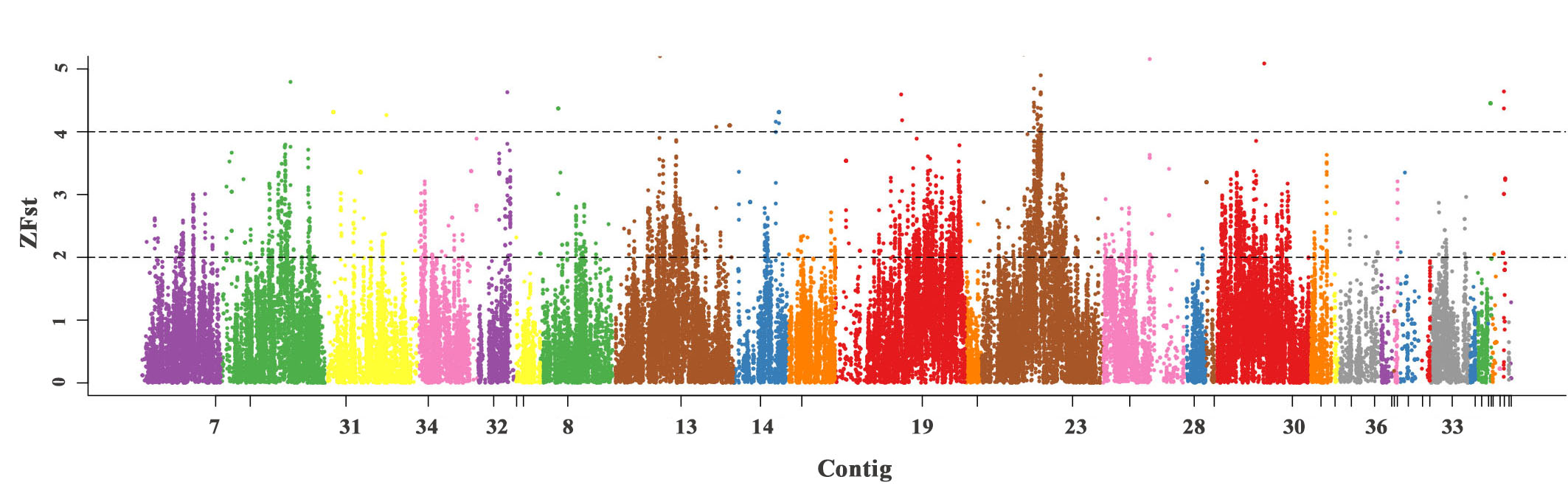

Supplement: FIGURE S1 — The genome selection region of P. eryngii var. eryngii and var. ferulae populations using the referencing genome PEE81. [file Image_1.JPEG]
